# LAPAROSCOPIC CHOLECYSTECTOMY VERSUS MINILAPAROTOMY IN CHOLELITHIASIS: SYSTEMATIC REVIEW AND META-ANALYSIS

**DOI:** 10.1590/S0102-67202014000200013

**Published:** 2014

**Authors:** Paula Marcela Vilela CASTRO, Denise AKERMAN, Carolina Brito MUNHOZ, Iara do SACRAMENTO, Mônica MAZZURANA, Guines Antunes ALVAREZ

**Affiliations:** Centro Universitário Lusíada - UNILUS and Departamento de Cirurgia Geral do Hospital Guilherme Álvaro (Lusiada University Center - UNILUS and Departament of General Surgery, Guilherme Álvaro Hospital), Santos, SP, Brazil

**Keywords:** Cholecystectomy, Laparoscopy, Minilaparotomy, Systematic review

## Abstract

**Introduction:**

A introdução da técnica laparoscópica em 1985 foi um fator importante na colecistectomia por representar técnica menos invasiva, resultado estético melhor e menor risco cirúrgico comparado ao procedimento laparotômico.

**Aim:**

To compare laparoscopic and minilaparotomy cholecystectomy in the treatment of cholelithiasis.

**Methods:**

A systematic review of randomized clinical trials, which included studies from four databases (Medline, Embase, Cochrane and Lilacs) was performed. The keywords used were "Cholecystectomy", "Cholecystectomy, Laparoscopic" and "Laparotomy". The methodological quality of primary studies was assessed by the Grade system.

**Results:**

Ten randomized controlled trials were included, totaling 2043 patients, 1020 in Laparoscopy group and 1023 in Minilaparotomy group. Laparoscopic cholecystectomy dispensed shorter length of hospital stay (p<0.00001) and return to work activities (p<0.00001) compared to minilaparotomy, and the minilaparotomy shorter operative time (p<0.00001) compared to laparoscopy. Laparoscopy decrease the risk of postoperative pain (NNT=7) and infectious complications (NNT=50). There was no statistical difference between the two groups regarding conversion (p=0,06) and surgical reinterventions (p=0,27), gall bladder's perforation (p=0,98), incidence of common bile duct injury (p=1.00), surgical site infection (p=0,52) and paralytic ileus (p=0,22).

**Conclusion:**

In cholelithiasis, laparoscopic cholecystectomy is associated with a lower incidence of postoperative pain and infectious complications, as well as shorter length of hospital stay and time to return to work activities compared to minilaparotomy cholecystectomy.

## Abstract (pt)

**Objetivo:**

Comparar a colecistectomia laparoscópica e a minilaparotômica no tratamento da colecistolitíase.

**Métodos:**

Realizou-se busca eletrônica nas bases de dados Medline, Embase, Cochrane e Lilacs. Os descritores utilizados foram “Cholecystectomy”, “Cholecystectomy, Laparoscopic” e “Laparotomy”. A qualidade metodológica dos estudos primários foi avaliada pelo sistema Grade.

**Resultados:**

Foram incluídos dez ensaios clínicos randomizados, totalizando 2043 pacientes, sendo 1020 no grupo Laparoscopia e 1023 no grupo Minilaparotomia. A colecistectomia laparoscópica dispensou menor tempo de permanência hospitalar (p<0,00001) e de retorno às atividades laborais (p<0,00001) comparado à minilaparotomia, e esta menor tempo cirúrgico (p<0,00001) comparado à laparoscopia. A laparoscopia diminuiu o risco de dor pós-operatória (NNT=7) e de complicações infecciosas (NNT=50). Não houve diferença estatística entre os dois grupos em relação à conversão (p=0,06) e reintervenções cirúrgicas (p=0,27), perfuração da vesícula (p=0,98), incidência de injúria do ducto biliar comum (p=1,00), infecção de sítio operatório (p=0,52) e íleo paralítico (p=0,22).

**Conclusão:**

Na colecistolitíase, a colecistectomia laparoscópica está associada à menor incidência de dor pós-operatória e complicações infecciosas, assim como menor tempo de internação hospitalar e tempo de retorno às atividades laborais se comparada à colecistectomia minilaparotômica.

## INTRODUCTION

Cholelithiasis is one of the most common digestive tract diseases and constitutes an important health problem in developed countries. It is estimated that 10-15% of the adult population accounting for 20 to 25 million americans have or will have gallstones^13^. The third National Health and Nutrition Assessment estimates that 6.3 million of men and 14.2 millions of women aged between 20 and 74 years in the United States had gallbladder disease^7^. Besides the problems related to health, cholelithiasis also brings significant costs, estimated at around 6.2 million dollars annually in the same country^13^.

About 750,000 patients undergo cholecystectomy per year in the United States, and the number of surgical procedures has grown increasingly over the years, with the purpose to avoid the symptoms, complications and recurrence of gallstones in the biliary tract^13^.

In Brazil, cholelithiasis is the most common abdominal surgical disease in elderly patients, its incidence being associated with the progression of age, with an overall prevalence in the general population of 9.3%^1^. In the last two years in the Unified Health System, according to the Datasus, conventional cholecystectomy represents 88% of the surgeries, compared to 12% of laparoscopic cholecystectomy^3^, this probably explains why the distribution of equipment and offer of services related to the procedure is quite uneven, being the most modern techniques adopted in a limited number of countries, and most of these have neither the technology nor the qualified professional for this procedure, but in the private sector in Brazil, it is clear an absolute predominance of laparoscopic cholecystectomy over the conventional cholecystectomy during the whole period (90% or more of total)^1^.

The introduction of the laparoscopic technique in 1985, first made by Mühe was an important factor for the large increase in the cholecystectomy , since it represented a less invasive technique, generated better esthetic result and provided a lower surgical risk compared to the conventional procedure^17^.

Dubois and Barthelot introduced in 1982, minimally invasive technique for conventional cholecystectomy, the minilaparotomy cholecystectomy^6^, and Tyagi et al, in 1994, described a new technique for minimally invasive cholecystectomy, and this has recently challenged the role of laparoscopic cholecystectomy^23,8^.

This review aims to compare laparoscopic and minilaparotomy cholecystectomy in the treatment of cholelithiasis regarding perioperative complications, length of hospital stay, surgical time, incidence of reoperation and conversion to open surgery and time for returning to labor activities.

## METHODS

### Identification and selection of studies

A search of electronic literature was done through the data bases MEDLINE, EMBASE, COCHRANE, and LILACS. On Medline and Embase the combination of terms (Cholecystectomy) and (Cholecystectomy, Laparoscopic) and (Laparotomy) were utilized. On LILACS and Cochrane, the keywords used were: (Cholecystectomy) and (Laparoscopy) and (Laparotomy). Manual searches were done among study references found. The searches ended on July 5, 2013.

The articles were selected independently and in pairs, by reading the titles and abstracts. Any difference between the articles was resolved by consensus.

### Inclusion and exclusion criteria 

Inclusion criteria: 1) randomized controlled trials; 2) comparison between laparoscopic and minilaparotomic cholecystectomy in cholelithiasis; 3) analysis of at least one of the outcomes described below; 4) a clear description of the surgical indication.

Exclusion criteria: 1) non-randomized trials, cohort, case-control and case report; 2) outcomes of interest not reported for both surgical techniques; 3) failure to provide data for performing at least one calculation in the meta-analysis; 4) studies that correspond to the same sample and identical study authors.

### Outcomes analyzed

They were length of hospital stay, operative time, surgical conversion, reoperation, time to return to labor activity and perioperative complications, divided into: 1) intraoperative complications (perforation of the gallbladder and common bile duct injury); and 2) postoperative complications (surgical site infection, pain, postoperative ileus, infectious complications).

### Methodological quality and statistical analysis

The methodological quality of the primary studies was evaluated by the GRADE system proposed by the Grades of Recommendation, Assessment, Development and Evaluation group.^4^

The meta-analysis was performed with the Review Manager 5.2 program. Data were evaluated by intention-to-treat, meaning the patients that did not undergo the proposed intervention or patients lost in follow-up during the study were considered as clinical outcome.

The evaluation of the dichotomic variables was performed by the difference in absolute risk (RD) adopting a 95% confidence interval. When there was a statistically significant difference between the groups, the number needed to treat (NNT) or the number needed to cause harm (NNH) was calculated. The continuous variables were evaluated by the difference in means (MD). Studies that did not show data in terms of means and their respective standard deviations were not included in the analyses.

### Heterogeneity and sensitivity analysis

Inconsistencies among the clinical studies were estimated using the chi-squared heterogeneity test and quantified using I^2^. A value above 50% was considered substantial. Studies that generated heterogeneity were represented by funnel plots.

## RESULTS

### Study selection

**Figure 1:**
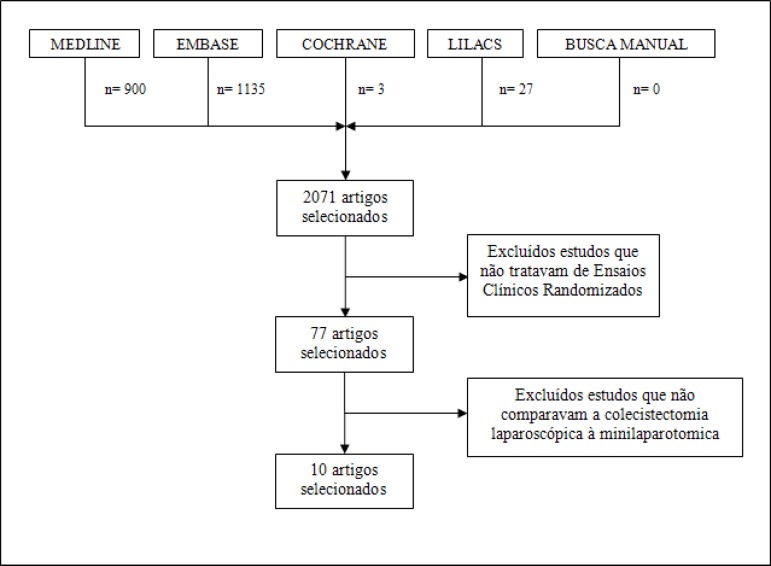
Search algorithm of articles in the literature

In total, 2071 articles (Medline=900; Embase=1135; Cochrane=3 and Lilacs= 27) were retrieved through the electronic searches. In manual search no articles were found in addition to the previously selected on the bases cited. After using methodological filter Randomized Controlled Trial, 77 articles remained for analysis by title and abstracts. Sixty-seven were excluded for not comparing laparoscopic and minilaparotomy cholecystectomy. Thus, in this review were included in the analysis ten randomized clinical trials (Figure 1).

### Description of included studies

The ten studies included randomized patients into two groups, laparoscopy and minilaparotomy, totaling 2043 patients, 1020 in the laparoscopy group and 1023 in the minilaparotomy group (Table 1).

**Table 1:** Description of included studies

Name	Year of publication	Patients (n)	Laparoscopy	Minilaparotomy	Outcomes
Velázquez-Mendoza^25^	2012	88	43	45	Bleeding; surgical site infection; perforation of the gallbladder; hepatic injury; postoperative ileus; postoperative pain
Harju^10^	2010	60	31	29	Infection; common bile duct injury; bleeding
Keus^12^	2007	257	120	137	Inflamation; abscess; pancreatitis; common bile duct injury; fistula; time to return to labor activity
Vagenas^24^	2006	88	44	44	Fever; hernia; pneumonia; postoperative ileus; time to return to labor activity
Harju^9^	2006	157	72	85	Surgical site infection; pneumonia; ITU; fistula
Srivastava^22^	2001	100	59	41	Fistula; surgical site infection; wound with pus; pain
Ros^19^	2001	724	362	362	Bleeding; pancreatitis; surgical site infection; thromboembolism; pulmonar infection; time to return to labor activity; cardiac complication; fistula; postoperative pain; perforation of the gallbladder, bleeding, vascular injury, intestinal injury, hepatic injury
Majeed^15^	1996	200	100	100	Subphrenic collection; surgical site infection; urinary retention; bile duct injury; chest infection; time to return to labor activity
McMahon^16^	1994	302	151	148	Infection; hematoma; urinary retention; ITU; septicemia; incisional hernia; IAM; chest infection; fistula; pain; brida's obstruction; postoperative bleeding
Barkun^2^	1992	70	38	32	surgical site infection; perforation of the gallbladder; postoperative ileus; pain

ITU=urinary tract infection; IAM=acute myocardial infarction

### Methodological quality

Evaluation of methodological quality of the selected studies performed by GRADE system include 11 questions that were answered as: Y=yes; N=no; ND=not described (no information enabling the evaluation).

The questions and answers according to each study were: 1) was the study randomized? Y for all; 2) was the allocation of patients to groups confidential? N to Velázquez-Mendoza (2012) and Y for the rest; 3) were patients analyzed in the groups to which they were randomized (was the analysis by intention to treat)? Y for all; 4) were patients in both groups similar with respect to the previously known prognostic factors? Y for all; 5) was the study blind? ND to Vagenas (2006) Harju (2006), Srivastava (2001), McMahon (1994) and Barkun (1992) and Y for the remainder; 6) except the experimental intervention, were the groups treated equally? Y for all; 7) were the losses significant? ND to Vagenas (2006) Harju (2006) and Srivastava (2001) and N for the remaining; 8) did the study have a precision estimate for the effects of treatment? Y for all; 9) are the study patients similar to those of interest? Y for all; 10) are the outcomes of the study clinically relevant? Y for all; 11) were the potential conflicts of interest declared? ND for all.

### Outcomes analyzed

#### Length of hospital stay

**Figure 2:**
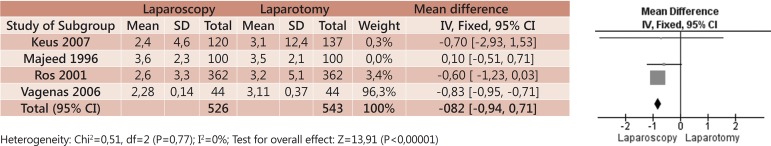
Meta-analysis of the mean difference in length of hospital stay between laparoscopy and minilaparotomy in patients with cholelithiasis

Four studies analyzed the primary outcome length of hospital stay; however, due to the high heterogeneity (MD -0,79 CI95% -0,90 a -0,68; p<0,00001 e I^2^=67%) related to the study of Majeed (1996), was chosen to exclude it from the analysis. The new forest-plot showed a mean difference between groups of 0.82 (CI95% -0,94 a -0,71; p<0,00001 e I^2^=0%). Thus, laparoscopy dismissed shorter hospital stay compared to minilaparotomy (Figure 2).

#### Surgical time

**Figure 3:**
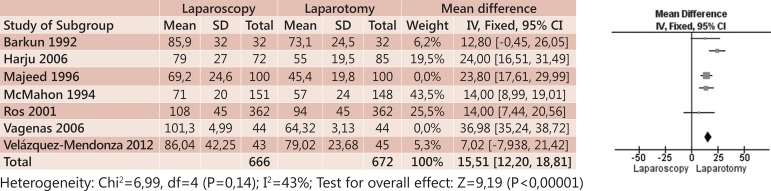
FIGURE 3 - Meta-analysis of the mean difference in surgical time between laparoscopy and minilaparotomy in patients with cholelithiasispacientes com colecistolitíase

Seven primary studies analyzed the outcome surgical time; however, the studies Majeed (1996) and Vagenas (2006) promoted high heterogeneity (MD 31,83; CI95% 30,33 a 33,32; p<0,00001 e I^2^=96%) and were excluded from the initial forest-plot. Thus, the difference in mean between groups was 15.51 (CI95% 12,20 a 18,81; p<0,00001 e I^2^=43%), and that the minilaparotomy dismissed shorter surgical time compared to laparoscopy (Figure 3).

#### Surgical conversion

Nine primary studies analyzed the outcome surgical conversion. The conversion was defined: laparoscopy-laparotomy and minilaparotomy-laparotomy. There was no statistically significant difference between the two groups (RD -0,03; CI95% -0,06 a 0,00; p=0.06; I^2^=66%).

#### Surgical reintervention

Three primary studies analyzed the surgical reintervention. There was no statistically significant difference between the two groups (RD -0,01; CI95% -0,02 a 0,01; p=0.27; I^2^=0%).

#### Time to return to labor activity

**Figure 4:**
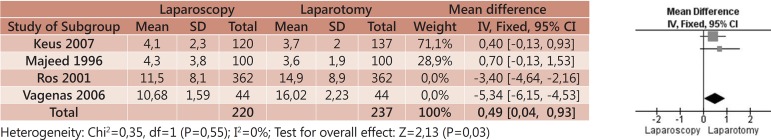
Meta-analysis of the time to return to labor activity between laparoscopy and minilaparotomy in patients with cholelithiasispacientes com colecistolitíase

Four studies analyzed the primary endpoint time to return to labor activity; however, studies of Ros (2001) and Vagenas (2006) showed high heterogeneity (MD 1,11; CI95% 0,73 a 1,48; p<0,00001 e I^2^=98%) being excluded from the analysis. Thus, the mean between groups was 0.49 (CI95% 0,04 a 0,93; p=0.03 e I^2^=0%) demonstrating that laparoscopy dismissed less time to return to labor activity compared to minilaparotomy (Figure 4).

### Intraoperative complications

#### Gallbladder perforation

Three primary studies analyzed the gallbladder perforation; however, the study of Ros (2001) promoted high heterogeneity (RD 0,11; CI 95% 0,06 a 0,16; p<0,0001; I^2^=91%), being excluded from the analysis. In the construction of the new forest-plot can be seen that there was no statistically significant difference between the two groups (RD -0,00; CI95% -0,05 a 0,05; p=0.98; I^2^=13%).

#### Injury to the common bile duct

Four primary studies analyzed the outcome common bile duct injury. There was no statistically significant difference between the two groups (RD 0,00; CI95% -0,01 a 0,01; p=1,00; I^2^=0%).

### Postoperative complications

#### Infection of the surgical site

Eight primary studies examined the infection of operative site; however, the study of Srivastava (2001) cause high heterogeneity (RD -0,02; CI95% -0,04 a -0,00; p=0,04; I^2^=61%). Thus, excluding this study from the analysis, there was no statistically significant difference between the two groups (RD -0,01; CI95% -0,03 a 0,01; p=0,52; I^2^=0%).

#### Postoperative pain

**Figure 5:**
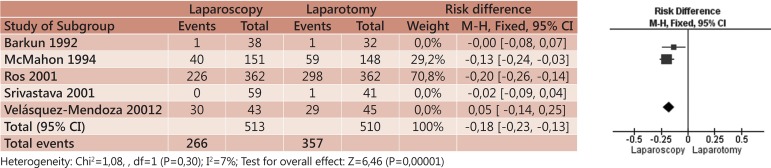
Meta-analysis of the incidence of postoperative pain between laparoscopy and minilaparotomy in patients with cholelithiasis

Five primary studies analyzed the postoperative pain. Three caused high heterogeneity (RD -0,14; CI95% -0,19 a -0,10; p<0,00001; I^2^=88%) to the analyzes (Barkun, Srivastava and Velázquez-Mendoza) and were excluded. Thus, the new forest-plot showed that laparoscopy reduced the absolute risk of post-operative pain in 18% (CI95% -0,23 a -0,13; p<0,00001; I^2^=7%; NNT=5) (Figure 5).

#### Postoperative ileus

Five primary studies analyzed the incidence of postoperative ileus. There was no statistically significant difference between the two groups (RD -0,01; CI95% -0,01 a 0,06; p=0,22; I^2^=0%).

#### Infectious complications

**Figure 6:**
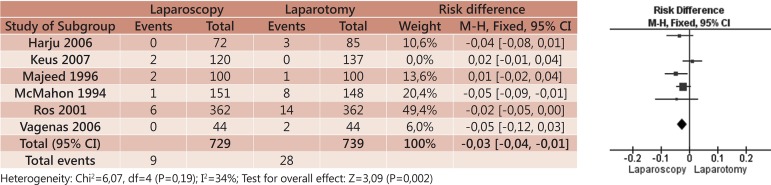
Meta-analysis of the incidence of infectious complications between laparoscopy and minilaparotomy in patients with cholelithiasis

Six primary studies analyzed the incidence of infectious complications (unrelated to the operative site); however, the study of Keus (2007) promoted high heterogeneity (RD -0,02; CI95% -0,03 a -0,00; p=0,009; I^2^=61%). Excluding this study from the analysis, it was observed that laparoscopy reduced the absolute risk of infectious complications in 3% (CI95% -0,04 a -0,01; p=0,002; I^2^=34%) (Figure 6).

## DISCUSSION

The first open cholecystectomy was performed by Carl Langenbuch in 1882, who believed in the theory that the gallbladder needed to be removed not because it had gallstones, but because it was "sick". After that, the technique was popularized through large incisions^12,24^. In 1985, Erich Mühe in Böblingen, Germany, performed the first laparoscopic cholecystectomy (LC), which became dominant process in the treatment of cholecystitis in late eighties^25^.

On the other hand, the advent of thin gauge surgical instruments and paradigms of minimally invasive surgery resulted in a gradual reduction in the length of incisions in the abdominal wall to open cholecystectomy. Subcostal oblique incision smaller than 8 cm in length is defined as minilaparotomy^21^. Can be performed with conventional surgical instruments available in any operating room; is slowly gaining acceptance as a low cost alternative compared to LC.^20^ Moreover, minicolecistectomy (MC) can be more profitable than LC because it eliminates the need for sophisticated equipment and specific medical staff ^24^.

The incidence of gallstones - one of the most important cause of morbidity in the world - should increase in next years due to obesity and older age, known risk factors in the development of cholelithiasis^10^. Currently, minimally invasive procedures, LC and MC, have largely replaced the procedure previously employed, the traditional cholecystectomy.^12^ However there are discussions about the advantages and disadvantages of minilaparotomy surgery in relation to laparoscopic ^8^.

The systematic review of Purkayastha *et al*^18^ (2007), that compared the LC and MC, included nine randomized controlled trials with a total sample of 2032 patients. All outcome measures had no statistically significant results, with the exception of surgical time and hospital stay (p<0.0001). Purkayastha showed that the mean of surgical time was 14.14 minutes higher in the group that performed the LC, and mean of hospitalization time was 0.37 days higher in the group that made ​​the MC. Comparatively, in this review we found that the mean of operative time was 31.83 minutes higher in the LC, and the mean of hospitalization was 0.79 days higher in the group that performed the MC.

In general, the Purkayastha's study demonstrated statistically significant results only for the conversion rate, abdominal complications and duration of sick leave (p=0.02, p=0.006 and p=0.009 respectively). The inability to perform meta-analysis of the costs of surgical procedures and analgesics requirements as well as aesthetic and quality of life - due to inconsistencies in the way that these results were reported - also limited conclusions that could be drawn.

In this review, it became clear that the LC showed a lower incidence of postoperative pain (p<0.00001). Patients' expectations and sociocultural influences are important additional factors that influence the use of analgesics. However, any cultural divergence on the consumption of drugs should affect both groups (LC and MC) equally^24^. In relation to infectious complications, they were less in the LC than MC (p=0.002).

The time to return to labor activity was lower in LC than in MC (p=0.03). The main determinants in this sense are subjective and influenced by the attitudes of patients and doctors^15^. According to Majeed *et al*^14^, surgeons and clinicians tend to keep patients undergoing MC out of work more than those who underwent LC. However, in this study, patients decided their time of sickie, and those undergoing MC returned to work at the same time or earlier than those who underwent LC.

Surgical conversion got no statistically significant result (p=0.06). It should be consider that the conversion of an LC or MC will not necessarily lead to a worse outcome patient^15^. The incidence of surgical site infection (p=0.52), injury to the common bile duct (p=1,00), perforation of the gallbladder (p=0.98), postoperative ileus (p=0.22) and surgical intervention (p=0.27) were not significant.

Purkayastha^18^ used in their meta-analysis, in certain outcomes, the odds ratio (OR) which shouldn't be used in therapeutic studies, because it distorts the veracity of the data and its heterogeneity. In this review, was chosen to express the results in the form of NNT or NNH when the data were statistically significant, which express respectively the required number of patients who need to be treated to obtain benefit or harm of the outcome analyzed.

Systematic review and meta-analysis is a type of study of scientific accuracy for selecting the best available evidence in the medical literature; but should also assess the methodological quality of primary studies. This is critical to obtaining accurate conclusions about the effect of interventions.^6^ To avoid distortions, it was decided to include only results with clinical and statistical homogeneity.

In this review was not used the Jadad^3^ scale for critical assessment of the methodological quality of primary studies, because it includes the blinding parameter. It is known that in surgical studies, particularly those that compare laparoscopic and laparotomic techniques, it isn't possible the blinding of the surgeon. Thus, the maximum Jadad scale in this type of study would be three, which would limit the selection of included studies.

The GRADE method includes the Jadad parameters and analyzes the most widely prognostic factors previously known, the estimation accuracy for the treatment effects, the similarity between the groups of patients, the clinical relevance of outcomes and the declaration of conflicts of interest.

One possible source of bias may be the differences between the processes of randomization of the included studies. However, the quality of the allocation process was considered adequate in all studies. All the patients analyzed had defined eligibility criteria.

In statistical analysis, the calculation of sample size and analysis by intention to treat were used. A common limitation of the analysis of surgical time and length of hospital stay was the lack of statistical measures such as standard deviation or present continuous data as median and range. However, the main limitation is the precise definition of MC used in the studies analyzed, ranging from 3 to 10 cm incisions.^16,22^

The study followed all the ethical and confidentiality principles of information that are recommended for dealing with analysis of results already published in other articles, was not required formal approval from a research ethics committee.

## CONCLUSION

Laparoscopic cholecystectomy is associated with a lower incidence of postoperative pain and infectious complications, as well as shorter hospital stay and time to return to labor activity, compared to minilaparotomy cholecystectomy. However, laparoscopy has longer surgical time compared to minilaparotomy. There was no statistically significant difference in outcomes surgical conversion, surgical site infection, surgical reintervention, injury of the common bile duct, gallbladder perforation and postoperative ileus.
